# Rare Case of Pseudohypoparathyroidism With Normocalcemia Because of a Novel GNAS Mutation

**DOI:** 10.1210/jcemcr/luad088

**Published:** 2023-08-09

**Authors:** Goutami Mangu, Sonika Malik, Vijay Eranki

**Affiliations:** Department of Internal Medicine, Texas Tech University Health Sciences Center, Odessa, TX 79765, USA; Department of Endocrinology, Diabetes and Metabolism, Texas Tech University Health Science Center, Odessa, TX 79765, USA; Department of Endocrinology, Diabetes and Metabolism, Texas Tech University Health Science Center, Odessa, TX 79765, USA

**Keywords:** pseudohypoparathyroidism, parathyroid hormone, guanine nucleotide binding protein, alpha stimulating activity polypeptide pathogenic variants, Albright hereditary osteodystrophy

## Abstract

PTH resistance is characterized by hypocalcemia and hyperphosphatemia in the presence of elevated PTH concentrations, resulting in pseudohypoparathyroidism, which is subdivided into different types according to its different pathogenesis and phenotype. PTH receptor is the alpha subunit of stimulatory G protein (G_s_α)-coupled receptor. Pathogenic variants of GNAS gene, encoding for G_s_α, lead to reduced G_s_α function and PTH resistance. We report a patient with PHP type 1a, with no documented evidence of hypocalcemia, presenting with AHO phenotype and multihormone resistance to PTH, TSH, and GnRH. Her genetic testing showed a novel heterozygous pathogenic variants, a c.934T > G change in exon 11 in adenylate cyclase stimulatory G protein that has not been reported in the literature so far.

## Introduction

Pseudohypoparathyroidism (PHP) refers to a group of heterogeneous disorders defined by targeted organ (kidney and bone) unresponsiveness to PTH. It is an uncommon sporadic or inherited genetic disorder subdivided into several distinct entities (types Ia, Ib, Ic; type II) according to its different pathogenesis and phenotype. PTH functions to modulate calcium and phosphate homeostasis. PTH resistance is characterized by hypocalcemia and hyperphosphatemia in the presence of elevated PTH concentrations. G_s_α-subunit-coding adenylate cyclase stimulatory G protein (GNAS) pathogenic variants (PaVs) that lead to diminished Gsα expression and/or function result in Albright hereditary osteodystrophy (AHO), which is a clinical entity often associated with PHP type 1a. It can present with or without hormone resistance (ie, pseudohypoparathyroidism type Ia/Ic and pseudo-pseudo-hypoparathyroidism, respectively). We report a patient diagnosed as PHP1a by her pediatrician, with no documented evidence of hypocalcemia, presenting with AHO phenotype and multihormone resistance to PTH, TSH, and GnRH, characterized by primary hypothyroidism and primary amenorrhea. Genetic testing revealed a novel PaV, c.934T > G change in exon 11 in GNAS1, which has not been reported in the literature so far and adds to spectrum of genetic PaVs associated to PHP-1A/1C.

## Case Presentation

A 23-year-old female was evaluated in our endocrinology clinic for management of PHP with physical features consistent with AHO, associated with longstanding hypothyroidism and multiple family members with short stature and stout body habitus in different generations. At the time of our evaluation, she was on levothyroxine and vitamin D supplements for primary hypothyroidism and vitamin D deficiency, respectively, detected during the work up of AHO by her pediatric endocrinologist.

The patient had physical features typical of AHO, with short stature, obesity, round face, shortened fourth and fifth digits in bilateral hands, low hairline, dental anomalies, multiple café au lait spots on face, dorsum of hands and shoulders, and developmental delay. She also complained of self-resolving small skin lumps and associated pain in random locations that appear periodically. According to the patient's mother, she stopped growing at the age of 7 years with evidence of fused growth plates on a subsequent bone scan. In addition, a skeletal survey showed the osseous features of AHO. Pubarche, thelarche, and adrenarche began at age 11 years, but she had primary amenorrhea with failure of bleeding after progesterone withdrawal. A trial of oral contraceptives and metformin did not result in menstruation.

Laboratory testing showed a high PTH of 90 pg/mL (90 ng/L) (12-65 pg/mL [12-65 ng/L]), 25-OH D of 9 ng/mL (22.46 nmol/L) (20-40 ng/mL [49.9-99.8 nmol/L]), serum calcium of 8.8 mg/dL (2.2 mmol/L) (8.8-10.5 mg/dL [2.2-2.62 mmol/L]), phosphorus of 4.6 mg/dL (1.48 mmol/L) (3.1-5.5 mg/dL [1.0-1.7 mmol/L]), TSH of 11.9 µIU/mL (11.9 mIU/L) (0.7-5.7µIU/mL [0.7-5.7 mIU/L]), free T4 of 0.8 ng/dL (10.3 pmol/L) (0.6-1.9 ng/dL [7.7-24.5 pmol/L]), FSH of 4.3 mIU/mL (4.3 IU/L), LH of 2.4 mIU/L (2.4 IU/L), free testosterone of 3.4 pg/mL (0.11 nmol/L), estradiol of 33.7 pg/mL (123.6 pmol/L) (30-100 pg/mL [110-367 pmol/L]), prolactin of 6.5 ng/mL (6.5 µg/L), anti-thyroglobulin antibodies of <20.0 IU/mL (<20.0 kIU/L) (<41 IU/mL [<41 kIU/L]), and anti-thyroid peroxidase antibodies of 28.6 IU/mL (0-34.9 IU/mL [0-34.9 kIU/L]), ruling out autoimmune hypothyroidism. Genetic testing by PCR detected PaVs in GNAS1. DNA sequencing of exon 1 through exon 13 revealed c.934T > G change in exon 11, which would be expected to result in a F312V (phe 312Val) amino acid substitution in the protein. Analysis of this protein ranked the change as “probably damaging,” and the Sorting Intolerant from Tolerant program predicted such amino acid substitution and protein change would not be tolerated and would be expected to be a disease-causing PaV.

The patient did not have documented evidence of hypocalcemia or hyperphosphatemia and her calcium and phosphorous levels were always within normal limits regardless of the PTH levels. PTH levels fluctuated between 90 and 300 pg/mL (90-300 ng/L) with concomitant normal serum calcium and phosphorus levels, proving evidence for PTH resistance that is observed in PHP 1A/1C. Her low LH and FSH with primary amenorrhea and primary hypothyroidism could be explained because of partial GnRH and TSH resistance, respectively, both of which are known to be associated with PHP.

## Treatment

We treated her medically with ergocalciferol 50 000 units 3 times per week and levothyroxine 125 mcg/d and her PTH and TSH normalized, and vitamin D levels improved.

## Outcome and Follow-up

The patient continued to follow up with our clinic, remained adherent to medical management, and on request was referred to gynecology for fertility treatment.

## Discussion

PTH functions to modulate calcium and phosphate homeostasis. It increases serum calcium, decreases serum phosphate, and stimulates the production of 1,25-(OH)_2_-vitamin D (calcitriol). PTH mobilizes calcium from skeletal stores and stimulates bone resorption and renal calcium reabsorption as well as interacting with calcitriol to increase intestinal calcium and phosphate absorption. In pseudohypoparathyroidism, the end-organ (kidney and bone) is resistant to PTH, resulting in hypocalcemia and hyperphosphatemia. Consequently, the normally functioning parathyroid glands further increase the PTH secretion causing hyperparathyroidism.

PTH receptor is a G_s_α-coupled receptor that, when stimulated, activates adenylyl cyclase and mediates cAMP production, which further stimulates intracellular protein kinases. These intracellular signal transduction pathways are responsible for the physiologic actions of PTH. GNAS (20q13.32) is a complex imprinted gene locus that encodes G_s_α, the α-subunit of the stimulatory G protein. Different peptide hormones including CRH, ACTH, GHRH, LH/CG, FSH, TSH, and PTH function through G_s_α receptors. Loss-of-function of G_s_α or of genes encoding for proteins downstream of the signaling cascade result in hormonal resistance and cause a variety of signs and symptoms depending on parental origin, type (genomic or epigenetic), and location of the alteration [1].

Patients with *GNAS* (PHP-Ia) pathogenic variants seem to have an earlier onset and more marked phenotype than those with loss of imprinting (mild-PHP-Ia and PHP-Ib). The clinical features of AHO (particularly those related to weight and subcutaneous ossifications) are less pronounced in patients with loss of methylation (mild PHP-Ia) than in those who present structural PaVs in *GNAS* (classic PHP-Ia). Similarly, hypothyroidism is diagnosed earlier than PHP in patients with genetic sequence alterations compared with those with loss of imprinting, which explains the greater sensitivity to haploinsufficiency in thyroid than parathyroid cells in these patients [2].

Renal proximal tubule, thyroid, pituitary, and gonad lack G_s_α derived from the paternal GNAS allele; thus, maternally derived genomic loss-of-function PaVs cannot be compensated for and perhaps lead to resistance of PTH, TSH, GHRH, LH, and FSH [3]. In most tissues with biallelic (paternal and maternal) GNAS expression, a single heterozygous PaV does not interfere with sufficient gene function. However, growth plate chondrocytes require 2 functional copies of G_s_*α* for normal development. Accordingly, AHO features—short stature and shortened metacarpals and metatarsals—are possibly caused by haploinsufficiency of G_s_*α* in bone tissue independent of parental origin. Thus, maternal G_s_α PaVs lead to AHO with hormone resistance (ie, pseudohypoparathyroidism type-Ia), whereas paternal PaVs cause AHO alone (ie, pseudo-pseudohypoparathyroidism) [4].

Patients with PHP-Ia display resistance to PTH and other hormones such as TSH, gonadotropins, and GHRH that act via Gs-coupled receptors, in variable severity. They show a reduction in the phosphaturic response to PTH, thus leading to hyperphosphatemia. Typical electrolyte disarray seen with PHP-Ia is hypocalcemia resulting from hyperphosphatemia and renal resistance to PTH, leading to a defect in the generation of 1,25(OH)_2_D [5]. However, anticalciuric action of PTH on the kidneys is conserved in all patients with PHP in contrast to phosphaturic effects at the proximal tubule of the kidney. Hence, these patients have normohypocalciuria without increased risk of kidney stones or abnormal renal function (Table 1).

**Table 1.** Pseudohypoparathyroidism types

Type	AHO	Hormonal resistance	GNAS defect
PHP-Ia	+	Multiple: PTH, TSH, GHRH, Gn	Maternal inactivating PaVs
PPHP	+	None	Paternal inactivating PaVs
PHP-Ib	−	PTH, TSH	Imprinting defect
PHP-Ic	+	Multiple: PTH, TSH, Gn	Few inactivating PaVs reported

Abbreviations: AHO, Albright hereditary osteodystrophy; GHRH, growth hormone releasing hormone; Gn, gonadotropin; GNAS, guanine nucleotide binding protein, alpha stimulating activity polypeptide; PHP, pseudohypoparathyroidism; PPHP, pseudo-pseudohypoparathyroidism.

PHP-Ia and PHP-Ic share common features of AHO and multihormone resistance. PHP-Ia patients demonstrate partial deficiency of Gs activity (about 50%) in the membranes of various cell types (erythrocytes, fibroblasts, platelets), which is absent in patients with PHP-Ic [5].

What makes this case interesting is the presence of a novel missense PaV that we found associated with PHP. The c.934T > G change in exon 11, which would be expected to result in a F312V(phe 312Val), has not been previously cited in the literature and adds to the spectrum of genetic PaVs associated to PHP-1A/1C. PaVs reported in exon 11 of GNAS gene so far were gene deletion leading to frameshift PaVs and, more recently, a novel heterozygous nonsense PaV, c.2787_2788del (p.Val930AspfsTer12), in exon 11 of the GNAS gene in a Chinese population has been reported, which is again a gene deletion leading to frameshift variants [6]. In a recent review, 176 different germline PaVs in the 13 exons of *GNAS* gene that encode Gsα resulting in PHP-1a have been reported and PaVs affecting the carboxyl terminal of the G-protein encoded by exon 13 have been reported to be associated with PHP-Ic [7]. More recently, a novel heterozygous c.715A > G (p.N239D) mutation in exon 9 of the *GNAS* gene was identified in a young patient who had a very atypical presentation with normocalcemia [8].

We report a patient who presented with AHO phenotype with café au lait spots, PTH resistance and normocalcemia, elevated PTH, low vitamin D levels with response to vitamin D supplementation, resulting in normalization of PTH and 25 hydroxy vitamin D levels. Our patient had resistance to TSH and GnRH, explained by primary hypothyroidism and primary amenorrhea. Though we do not have phosphaturic response and urinary cAMP studies in our patient, with elevated PTH and multiple hormone resistance explains the electrolyte disarray and phenotypical features are due to GNAS PaV syndromes than vitamin D deficiency alone. Genetic testing revealed a novel c.934T > G change detected in exon 11 that has not been reported in literature so far. This missense PaV results in a F312V (phe 312Val) change. However, whether this pathogenic genetic variant was maternally or paternally inherited could not be determined. Testing for G_s_α activity in erythrocytes could not be done to differentiate from PHP-Ic because of financial constraints but with the presence of the GNAS PaV in exon 11, the patient was diagnosed as PHP-IA.

## Learning Points

- Pseudohypoparathyroidism can present with normocalcemia and multihormone resistance
- GNAS PaVs are associated with AHO phenotype, resistance to TSH, GnRH, GH along with PTH
- Novel PaV detected in exon 11, c.934T > G change, adds to spectrum of pathogenic genetic variants associated to PHP-1A/1C

## Contributors

G.M. was involved in writing and drafting the case report. V.E. and S.M. were involved in the diagnosis and management of the patient and helped review the case report. All authors contributed to the authorship and reviewed and approved the final draft.

## Data Availability

Original data generated and analyzed during this study are included in this published article.

## Abbreviations

AHO: Albright hereditary osteodystrophy
GNAS: G_s_α-subunit-coding adenylate cyclase stimulatory G protein
PaV: pathogenic variant
PHP: pseudohypoparathyroidism
